# Vegetation change threatens the sustainability of cultural keystone species and traditional ecological knowledge in a social–ecological system

**DOI:** 10.1038/s41598-025-24015-6

**Published:** 2025-11-17

**Authors:** Shiori Takahashi, Jun Nishihiro

**Affiliations:** 1 Graduate School of Agricultural and Life Science, The University of Tokyo, 1-1-1, Yayoi, Bunkyo-ku, Tokyo, 113-8657 Japan; 2 Center for Climate Change Adaptation, National Institute for Environmental Studies, 16-2 Onogawa, Tsukuba, Ibaraki 305-0053 Japan

**Keywords:** Biocultural diversity, Cultural keystone species, Local ecological knowledge, Marsh vegetation, Social-ecological systems, Vegetation monitoring, Environmental social sciences, Sustainability, Conservation biology

## Abstract

Traditional ecological knowledge (TEK) and cultural practices are often closely linked to local biodiversity through keystone species. However, the long-term ecological dynamics affecting the utility of such species remain underexplored. This study combines TEK with 40 years of vegetation monitoring in a Japanese wetland to examine how ecological change affects culturally important thatch. Interviews and sample observations categorized species into four ranks, from essential to undesirable for thatching. This index was then applied to analyze long-term vegetation monitoring data. Conventional indices such as species richness and the Shannon–Weaver index showed no clear trends, while NMDS detected shifts in species composition. However, these shifts were not directly linked to cultural usefulness. In contrast, the usefulness-based assessment revealed a substantial decline in species essential for high-quality thatch and an increase in undesirable species. This shift suggests a decline in thatch quality and a potential threat to the sustainability of the social–ecological system reliant on thatch use. Our findings demonstrate the value of integrating TEK with ecological monitoring to detect ecologically and culturally significant change. This interdisciplinary approach yields insights into social–ecological interactions that would be missed by standard metrics, and can inform context-sensitive management strategies to support system resilience.

**Supplementary Information:**

The online version contains supplementary material available at 10.1038/s41598-025-24015-6.

## Introduction

Many traditional cultures have been maintained by using plants or animals as material resources. Recent global studies have emphasized the importance of identifying species that hold high significance in shaping cultural identity and practices, defined as “cultural keystone species” (CKS;^1^). CKS encompass not only the species themselves but also involve complex interactions among traditional knowledge, techniques, and local community structures centered around these species^2^. Previous studies have identified CKS in various contexts, such as the néré tree (*Parkia biglobosa*) in West Africa, which sustains food, medicine, and social networks^3^, medicinal plants in Europe (Southern Alps and Spain), which link traditional healing practices with biodiversity conservation^4,5^, and the tagua palm (*Phytelephas aequatorialis*) in Ecuador, important in religious festivals and community traditions^6^. Understanding the sustainability of social–ecological systems (SESs) requires attention to the presence and condition of CKS, which are deeply embedded in both ecological functions and cultural practices. Identifying such species is therefore critical for evaluating SES dynamics. Ecological changes can alter cultural practices and management customs, potentially initiating feedback loops that contribute to destabilizing shifts in the SES.

Environmental changes, such as shifts in climate and land use, often lead to changes in the species composition of ecosystems, which in turn affect the viability of cultural systems. This may influence the persistence of traditional knowledge and cultural practices (e.g.,^7–11^). However, not all changes in species composition necessarily affect cultural sustainability^5,12^. We argue that identifying and analyzing CKS as culturally informed ecological indicators is essential to assess how changes in biological communities affect traditional practices, and we demonstrate this approach in our study.

Creating such indicators requires interdisciplinary research that integrates cultural practices with ecological assessments. Although the importance of interdisciplinary approaches to SES studies has been widely acknowledged^13^, ecological data are often underutilized compared with sociological data^14,15^. Many ecological studies have focused on the long-term monitoring of ecosystems, but their discussions have often remained limited to hypothesizing the cultural impacts of ecological changes, without extending to quantitative assessments (e.g.,^16,17^). Translating and utilizing ecological data from a cultural perspective is essential, yet such studies remain scarce (e.g.,^18,19^). What has been missing is a way to quantitatively link long-term ecosystem changes to changes in cultural practices. We hypothesized that by developing a culturally informed ecological index, we could detect changes in resource quality that conventional metrics overlook.

Here, we addressed this gap by developing indicators based on humanities-driven research to analyze the long-term vegetation changes in marsh ecosystems and their implications for resource availability within a cultural SES. To ground our study, we focused on a traditional Japanese grassland system known as *kayaba*, which exemplifies a SES centered on CKS. A *kayaba* is a wet or dry grassland, commonly managed for the harvesting of thatch. Historically, each village or household in Japan maintained its own *kayaba*, where grasses were cut for roofing, fodder, livestock bedding, and fertilizer^20^. Owing to the high demand for grass resources, mowing is conducted in areas dominated by Poaceae species useful for thatch, and is sometimes combined with controlled burning to improve both the yield and quality of thatch^21,22^. *Kayaba* was managed as a common by self-governing organizations under a labor exchange-centered mutual assistance system, and it functioned as a place that strengthened the social capital within local communities^23^. Following World War II and subsequent economic development, the use of *kayaba* declined, resulting in their progressive disappearance across Japan. The techniques of thatching and the harvesting of plants for thatch are listed as UNESCO Intangible Cultural Heritage for their cultural significance and uniqueness. Moreover, *kayaba* sites have been reported to support diverse flora and fauna characteristic of grasslands and wetlands, thus playing an important role in biodiversity conservation (e.g.,^24,25^). Globally, grasslands have declined as traditional management was abandoned, with ecological degradation closely intertwined with the erosion of cultural practices (e.g.,^26–28^). In Japan, changes in land use and lifestyles have led to the decline of semi-natural grasslands, resulting in the loss of both biodiversity and cultural values^29^. Although the decline of managed grasslands is not unique to Japan, *kayaba* represent a distinctive case where traditional practices of thatch harvesting have persisted and remain closely tied to cultural identity.

**Fig. 1:**
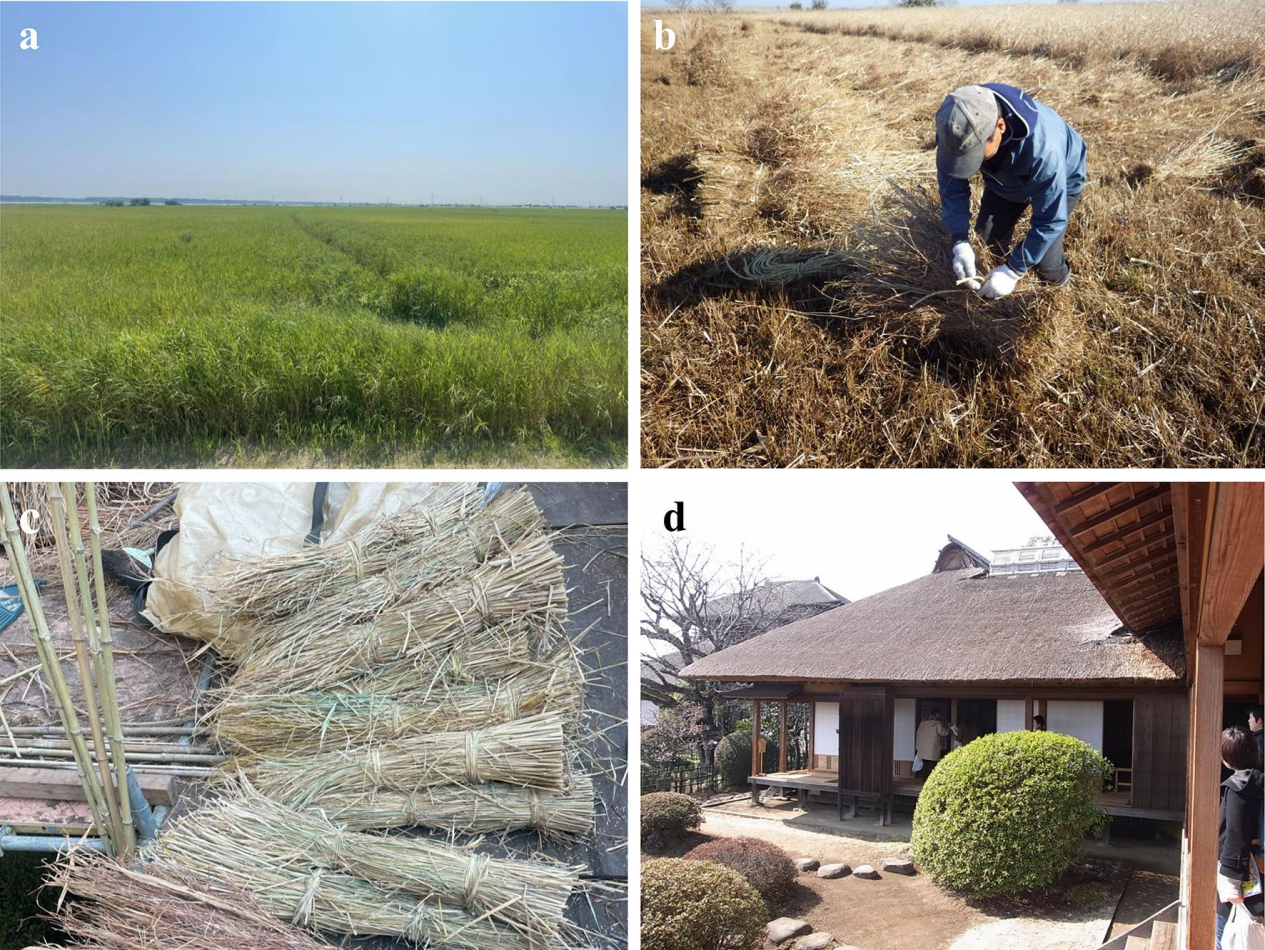
*Kayaba* at Myoginohana. (**a**) Summer view of the Myoginohana. (**b**) Harvesting of thatch conducted between December and January. (**c**) Thatch bundles of *Shimagaya*. (d) A cultural property building with a traditional thatched roof.

Our study focuses on Myoginohana Marsh, a well-known *kayaba* that has long been valued for its high-quality thatch (Fig. 1). The area maintained by the Japan Water Agency has more than 40 years of vegetation survey records. Through interviews with thatch users and observations of thatch samples, we categorized the plant species in this *kayaba* into ranks on the basis of their usefulness as thatching materials. We then used this classification as an indicator to analyze 40 years of ecological data and to thus assess the long-term changes in the species abundance within each rank. In light of these findings, we discuss how long-term ecological changes surrounding CKS affect cultural SES and propose effective management strategies to sustain the cultural SES of thatch use and enhance its resilience.

## Results

### Semi-structured interviews and observations of thatch samples

From the results of our interviews and observations of thatch samples, plant species were categorized into four ranks (A, B, C, and D) according to their usefulness as thatch materials (Table 1).

**Table 1:** Usefulness-based classification of species for thatch. List of usefulness of species for thatch, as determined from semi-structured interviews and from observations of unused recent thatch samples and old used thatch samples. Rank E species were not included in the analysis.

Rank	Classification	Plant species
A	Species useful as thatch	*Phragmites australis*, *Ischaemum aristatum* var. *glaucum*, *Arundinella hirta*, *Hemarthria sibirica*, *Miscanthus sacchariflorus*, *Calamagrostis epigeios*
B	Species mixed in with thatch but not removed	*Persicaria japonica*, *Triadenum japonicum*, *Lycopus* spp., *Eleocharis* spp., *Lysimachia fortunei*, *Iris (Iris ensata* var. *spontanea,* etc.*), Lythrum salicaria*, *Lythrum anceps*,* Lespedeza cuneata*
C	Species that are used as thatch, but that, as their proportion increases, decrease the thatch quality	*Isachne globosa*, *Carex dispalata*
D	Species to be removed because reduce the thatch quality	*Solidago altissima*, *Rosa multiflora*, *Rosa luciae*, *Paederia foetida*, vine plants
-	Species of unknown usefulness	Other than above

Both the thatcher and the thatch cutter identified *I. aristatum* var. *glaucum* as the most important species for making high-quality *shimagaya*. They emphasized that a high proportion of this species with long stems in each thatch bundle was essential for achieving high-quality *shimagaya*. Furthermore, they explained that they harvested mainly in areas where *I. aristatum* var. *glaucum* was present. Based on these characteristics, the species was classified as Rank A. Additionally, the thatcher identified *Phragmites australis* as another important species used in thatch (Table 2). Observations of thatch samples revealed that *I. aristatum* var. *glaucum* and *Arundinella hirta* were dominant in the old samples. Other Poaceae species, including *Hemarthria sibirica*, *Calamagrostis epigejos*, and *Miscanthus sacchariflorus*, were found in both old and recent samples and were classified as Rank A. These species were repeatedly mentioned as useful species by thatch users and were observed in old thatch samples, indicating their cultural and practical importance. Based on these findings, Rank A species could be recognized as culturally important for *shimagaya* production, and were collectively regarded in this study as CKS.

**Table 2:** Results of semi-structured interviews with the thatcher and the thatch cutter. Bold italic text indicates the common names of plant species mentioned during the interviews and is followed by the scientific names in parentheses.

Interview question	Response	
	Thatcher	Thatch cutter
What type of thatch is easy to use?	"Good thatch is long (about 1 m) and flexible, and when bent, it springs back." "When ***yoshi*** (*Phragmites australis*) is present in the thatch bundles, it makes them stronger." "The most important factor is ***kamonohashi*** (*Ischaemum aristatum* var. *glaucum*). The higher the proportion of ***kamonohashi***, the better the quality." "Additionally, straight stems with minimal bending are preferred." "***Ogi*** (*Miscanthus sacchariflorus*) is removed if it is bent, but otherwise, it is left as is."	"Harder and longer thatch is better. Soft ones are not good."
What type of thatch is difficult to use?	"Ideally, thatch bundles should be tied in a rocket shape. If there are too many leaves at the tip, the volume of the bundle’s head becomes too large, making it difficult to use. Removing excess leaves also requires extra effort." "Thatch that was not cut last year and is harvested this year becomes brittle and breaks easily, making it unsuitable for use." "If thatch is harvested before it has fully dried out, it becomes too soft and lacks the necessary firmness."	
Are there any species mixed in with the thatch that cause problems?	"Species such as ***seitaka*** (*Solidago altissima*), ***ibara*** (thorny plants), and ***tsuru*** (vine) species are problematic. Bent or vine-like species need to be removed because they interfere with the bundling process." "This year, ***chigozasa*** (*Isachne globosa*) increased in abundance, and as its quantity grows, its numerous leaves make it more difficult to handle."	
Are there any species that are acceptable even if mixed in with the thatch?	"***Shirobana-sakuratade*** (*Persicaria japonica*), ***hagi*** (*Lythrum* spp.), and ***nohanashoubu*** (*Iris ensata* var. *spontanea*) are left in because I find their presence charming." "*Shimagaya* contains various plant species, and having a few of these mixed in does not substantially affect the roof "Thatchers do not usually know exactly what plants are inside *shimagaya* or their names. Preferences regarding the degree of mixture and the amount of leaves vary by individual and region."	
How has *shimagaya* changed compared with in the past?	"Looking at old thatch, the amount of ***kamonohashi*** was completely different. The older thatch has a reddish hue. It also feels as though the length has gotten shorter."	"In the past, high-quality thatch grew abundantly. ***Kamonohashi*** was widespread throughout the areas. It was also thicker and harder. Now, ***kamonohashi*** is not the only species growing, so there are areas where it can be harvested and areas where it cannot." "(Pointing to *Carex dispalata*) I do not know the names of the grasses, but now there are finer and softer grasses. Softer thatch likely affects the durability of the roof. Thatchers clean it up and use it for roofing, but during harvesting, there is no time to sort it carefully."
How is *shimagaya* used for roofing?	"The harvested thatch is tied into bundles by using a binder machine. Before being used on the roof, the bundle is loosened once, and the base is aligned to make it even. As the *shimagaya* is already short and its base is durable, it is used without cutting much of the lower part."	

Rank B included species that were not primary thatch materials but did not affect the quality of bundles when present. Interviews with the thatcher identified *Persicaria japonica, Lythrum* spp., and *Iris ensata* var. *spontanea* as acceptable for inclusion in thatch bundles. These species were classified as Rank B, reflecting the thatcher’s intent to incorporate plants that naturally coexisted in the area. Additionally, our analysis of old and recent thatch samples revealed that species such as *Eleocharis* spp., *P. japonica*, *Lysimachia ortune*, *Triadenum japonicum*, *Lycopus* spp., and *Lythrum* spp. Were consistently present. These species were also classified as Rank B, supporting the interview findings that Rank B species were those that coexisted with Rank A species and did not negatively affect thatch quality.

Rank C included species that we categorized as unsuitable for thatch owing to their structural characteristics, such as high leaf content or the presence of soft, fine structures that compromised the quality of the thatch bundles. *Isachne globosa* and *Carex dispalata* were described by the thatcher as unsuitable for thatch owing to their high leaf content and fine, soft structures, respectively, which resulted in an overly soft thatch material. Both the thatcher and the thatch cutter expressed concerns about the increasing proportion of these species in recent years, noting that their abundance contributes to softer, lower-quality thatch bundles. Although both *I. globosa* and *C. dispalata* were found in both old and recent thatch samples, their proportions in the old samples were notably lower. This classification indicates that although these species were occasionally used for thatching, an increase in their coverage proportion negatively affected the overall quality of the material.

Finally, Rank D included species that were entirely unsuitable for thatching, such as crooked, thorny, or non-straight vines (e.g., *Solidago altissima*, *Rosa multiflora*, *Rosa luciae*, *Paederia foetida*), as mentioned in the interviews. These species were categorized as Rank D because they were removed during the preparation of the thatch bundles. As expected, no Rank D species were present in the old (used) thatch samples because they had been removed during roof construction, consistent with interview statements describing their deliberate exclusion.

### Changes in vegetation in Myoginohana Marsh over time

**Fig. 2:**
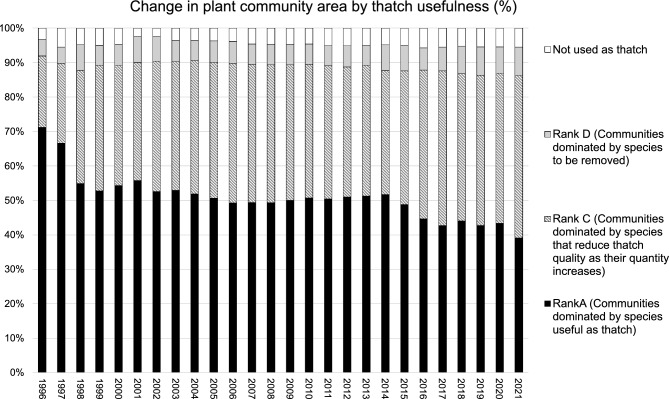
Proportion of each community’s coverage, reclassified by thatch usefulness and based on vegetation maps of Myoginohana Marsh from 1996 to 2021.

To understand how vegetation relevant to thatch use has changed over time, we reclassified vegetation maps based on the usefulness of plant species for thatching. The analysis focused on the period from 1996 to 2021, for which consistent data were available (Table 3). The total area dominated by Rank A species had decreased from 71.2 to 39.1%, whereas the area dominated by Rank C and D species had increased from 25.5 to 55.3% (Fig. 2).

**Table 3:** Correspondence between the plant communities identified in the monitoring survey in 1996 to 2021 and the reclassification of communities on the basis of their usefulness as thatch, for reanalysis of the vegetation maps.

Existing classification of plant communities	Rank	Reclassification of communities on the basis of usefulness as thatch
*Phragmites australis* (reed)	A	Communities dominated by species useful as thatch
*Ischaemum aristatum* var *glaucum* and reed	A	Communities dominated by species useful as thatch
*Miscanthus sacchariflorus*	A	Communities dominated by species useful as thatch
*Carex dispalata* and reed	C	Communities dominated by species that reduce thatch quality as their proportion increases
*Solidago altissima*	D	Communities dominated by species to be removed
Reed and *Solidago altissima*	D	Communities dominated by species to be removed
*Pueraria lobata* (kudzu)	D	Communities dominated by species to be removed
*Imperata cylindrica* (cogon grass)	D	Communities dominated by species to be removed
*Salix subfragilis* Andersson	-	Not used as thatch
*Celtis sinensis* var. *japonica*	-	Not used as thatch
Others	-	Not used as thatch

### Changes in vegetation, as determined from thatch quality

#### Comparison of conventional assessments and cultural-use-based evaluations

**Fig. 3:**
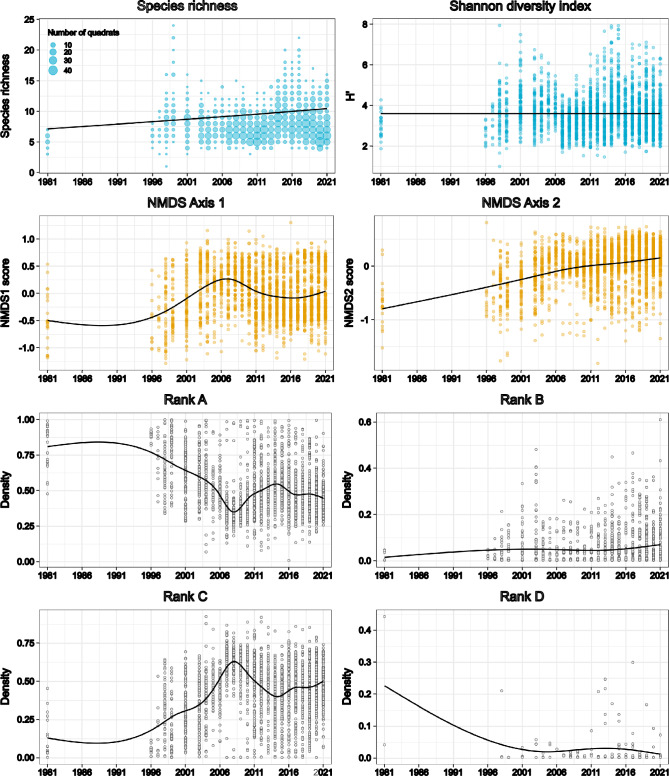
Temporal changes in species richness, Shannon diversity index, NMDS scores, and densities of Rank A to Rank D species within quadrats, analyzed using generalized additive model (GAM).

To assess long-term vegetation trends in the marsh, we applied a generalized additive model (GAM) to both conventional ecological indicators and a cultural-use-based ranking system. In our GAM analysis, the species richness in each quadrat showed an increasing trend between 1981 and 1996, with fluctuations observed in individual years (Fig. 3). However, no significant overall change was detected. A similar pattern was observed in the Shannon–Weaver diversity index. To further capture compositional changes, we performed a non-metric multidimensional scaling (NMDS) analysis. The ordination revealed a gradual temporal shift along NMDS2, which increased markedly after the late 1990s and was positively correlated with survey year (ρ = 0.36, p < 0.001), while NMDS1 showed only a weak negative correlation with year (ρ = –0.045, p = 0.025). PERMANOVA confirmed that these temporal changes were statistically significant (R^2^ = 0.163, F = 20.16, p = 0.001). Envfit analysis further identified several species strongly associated with these axes (Table 4). In particular, *Carex dispalata* and *Phragmites australis* (reed) were aligned with the positive direction of NMDS2, reflecting their increasing contribution after the 2000s, whereas *Isachne globosa* and *Ischaemum aristatum* var. *glaucum* were aligned with the negative direction, indicating that past communities in the 1980–1990s were characterized by these species. These associations indicate that NMDS captured temporal changes in species composition, but not necessarily the cultural usefulness of species. GAM revealed significant temporal trends in species richness (p = 0.0016) and NMDS1 (p < 0.001), whereas Shannon diversity showed no significant trend (p = 0.56). In contrast, our GAM analysis of the coverage proportions of the four ranks based on thatch usefulness revealed clear temporal changes. The overall trends indicated that, after 1996, the coverage proportion of Rank A substantially decreased, whereas that of Rank C substantially increased. Notably, Rank A and Rank C exhibited substantial changes in about 2008, which marked the lowest and highest points, respectively, for these ranks. In contrast, no marked temporal changes were observed for Rank B or Rank D.

**Table 4:** Species significantly associated with the NMDS ordination (k = 2) based on envfit analysis. Vector coordinates (NMDS1, NMDS2) and vector length indicate the direction and strength of association with ordination axes. “Rank” refers to the cultural-use-based classification system of thatch usefulness (A–C), with “–” indicating species not included in the ranking system.

Species	Rank	NMDS1	NMDS2	Length	q-value
*Isachne globosa*	C	−0.482	0.739	0.882	0.004**
*Carex dispalata*	C	0.866	0.038	0.867	0.004**
*Ischaemum aristatum* var.* glaucum*	A	−0.44	−0.449	0.628	0.004**
*Arundinella hirta*	A	−0.294	−0.452	0.539	0.004**
*Phragmites australis*	A	0.272	0.365	0.456	0.004**
*Miscanthus sacchariflorus*	B	0.043	−0.305	0.308	0.004**
*Triadenum japonicum*	B	−0.155	−0.057	0.165	0.004**
*Persicaria sagittata*	–	−0.058	−0.118	0.132	0.004**
*Platanthera hologlottis*	C	−0.065	−0.089	0.111	0.004**
*Fimbristylis complanata* (Retz.) Link	–	−0.07	−0.083	0.109	0.004**

q-values are adjusted using the Benjamini–Hochberg method.

**Indicates significance at q < 0.01.

Further examination of the main species trends within Rank A showed that *P. australis* displayed a slight increasing trend in coverage. *I. aristatum* var. *glaucum* showed a decreasing coverage trend from 1981 onward, reaching its lowest in 2007 and 2008, before showing a slight increase thereafter (Supplementary Fig. 1).

Both of the Rank C species *I. globosa* and *C. dispalata* exhibited increasing coverage trends, but with different patterns. The coverage of *I. globosa* gradually increased from 1981 to 2021. In contrast, *C. dispalata* demonstrated a substantial increase in coverage between 1996 and 2008, followed by a decline until approximately 2017. After 2018, its coverage began to increase again (Supplementary Fig. 1).

#### Assessment of seasonal changes in *I. aristatum* var. *glaucum* height

**Fig. 4:**
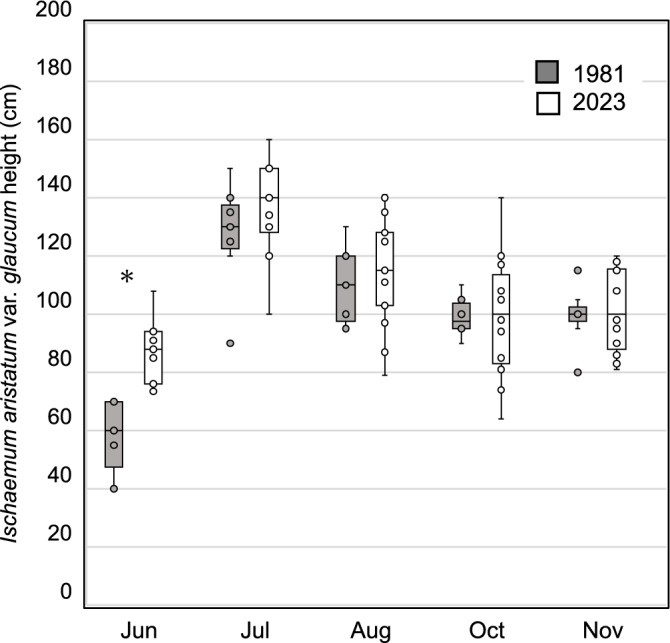
Monthly comparison of *Ischaemum aristatum* var. *glaucum* heights in 1981 and 2023. The asterisk indicates a significant difference (two-tailed *t*-test, *P* < 0.05).

To investigate whether the perceived decline in thatch quality is due to a reduction in the stem height of key species, we compared the seasonal height of *I. aristatum* var. *glaucum*, an essential thatching species, between 1981 and 2023. The height of *I. aristatum* var. *glaucum* in 2023 differed significantly only in June compared with the 1981 data (two-tailed *t-*test,* t* =  − 5.19, df = 16, *P* < 0.001), with no significant differences observed in the other months (Fig. 4).

## Discussion

Our results demonstrate that significant changes have occurred in the plant community composition of Myoginohana Marsh over the past several decades, with direct consequences for its value as a thatch resource. The ranking system established through interviews with thatch users revealed a clear trend: species classified as Rank A species decreased in coverage, while Rank C species, which diminish thatch quality, increased. This approach allowed us to evaluate the species in terms of their cultural importance for the use of thatch, based on both local knowledge and material evidence. Although the concept of CKS has been criticized for its limited objectivity and quantifiability^30^, our method provided a practical way to assess culturally important species in a specific social–ecological context.

Harvesting has traditionally been guided by local knowledge and practices. Among the Rank A species, *Ischaemum aristatum* var. *glaucum* is particularly important. It has historically been dominant in the Myoginohana Marsh. The interviews highlighted that local thatch users consider it a culturally important species due to its essential role in ensuring the quality of *shimagaya* for traditional thatching practices. The interviews also revealed that the harvesting sites are selected based on the presence of this species. In addition to its cultural value, *I. aristatum* var. *glaucum* is already known to act as a facilitator of biological interactions by forming small tussocks about 10 cm high that provide microsites less susceptible to inundation, thereby promoting seedling establishment^31^. These ecological and cultural roles underscore its function as a CKS that links ecosystem processes with human use. However, the area dominated by this species decreased from 61.1% in 1981 to 32.3% in 2021. Quadrat-level surveys further revealed a decline in its coverage, even in areas where the species persisted (Supplementary Fig. 1). This substantial decline in CKS represents a serious concern for ecosystem dynamics, the availability of high-quality thatch, and the continuity of traditional thatch use. A reduction in useful thatch species can diminish the incentive for mowing and burning practices, thereby weakening the management processes that have historically maintained the grassland ecosystems.

In contrast, Rank C species, including *Isachne globosa* and *Carex dispalata*, showed increased coverage over the same period. The coverage of *I. globosa* exhibited a gradual increase from 1981 to 2021, whereas that of *C. dispalata* showed fluctuations but had recently increased. Thatch is harvested using hand sickles or machinery, without distinguishing among plant species. It is then bundled for use. During roof construction, unwanted species are manually removed. Therefore, the increase in coverage of Rank C species indicates a decline in thatch quality, through both changes in composition and the increased handling costs required to process bundles. These results indicate that ecological change has contributed to a decline in thatch quality, leading to a reduction in the value of this *kayaba* as a thatch resource.

The factors that have driven the increase in coverage of Rank C species remain unclear, but the water level may be a key contributing factor. Both *I. globosa* and *C. dispalata* prefer wetter habitats and are commonly found at lower relative heights above the water surface in the Lake Kasumigaura region (Supplementary Fig. 2). Since the initiation of the water-level management regime in Lake Kasumigaura in 1996, water levels have remained approximately 20 cm higher than their previous levels. Previous studies have suggested that this increase may have affected the vegetation of Myoginohana Marsh^32–34^. The rise in water levels may have favored the Rank C species that thrive in wetter environments, thus altering the competitive dynamics among species. However, further studies, such as cultivation experiments, are needed to clarify the specific mechanisms by which environmental changes influence species composition.

Our interviews also highlighted the perception that *I. aristatum* var. *glaucum*, a key Rank A species, had shorter stems than in the past. However, our data comparing its height between 1981 and 2023 did not indicate a significant reduction in height. This discrepancy may be explained by the increased proportion of Rank C species in the bundles, which have shorter stems than *I. aristatum* var. *glaucum*. When many short-stemmed plants are present, the average stem length of a bundle is shorter than in the past, when long-stemmed species were dominant. This may contribute to the perception among thatch users that bundles have become shorter. These user perceptions, while not directly supported by the height data, were instrumental in guiding our investigation and highlight the value of local knowledge in detecting early signs of ecological change.

The shift in the balance between Rank A and Rank C species leads to reduced thatch quality and increased labor costs, posing a threat to the sustainability of the cultural SES associated with *the use of kayaba.* Indeed, the increased dominance of Rank C species in harvested areas already negatively impacts bundle quality and increases handling costs due to the increased labor required to remove leaves, as previously described in the interviews conducted for this study. Water-level change in the marsh has likely reduced the cover of the CKS, *I. aristatum* var. *glaucum*, thereby decreasing the availability of high-quality thatch. Such changes may trigger a concerning feedback loop: As high-quality thatch becomes scarce and more labor-intensive to use, remaining users may opt for alternatives such as thatch from other regions or imported materials, or they may even replace their thatched roofs with less maintenance-intensive materials.

This shift may weaken incentives for traditional management practices, such as cutting and burning, and the decline of these practices can negatively affect the growth of *I*. *aristatum* var. *glaucum*, which is known to benefit from such management^35^, leading to an accelerated loss of key thatch species and posing a threat to the long-term sustainability of *kayaba*. This process may initiate a concerning feedback loop, where reduced thatch quality and usability, declining user participation, and diminished management activities mutually reinforce each other, further weakening the cultural SES that supports the thatching tradition. The case of Myoginohana illustrates how changes in the abundance of CKS can trigger broader transformations in the social and cultural dimensions of a resource-dependent system.

One of the important findings of this study is that using the usefulness-based ranking system revealed changes in species composition that conventional ecological metrics (e.g., species richness, diversity indices, and NMDS) failed to capture. NMDS detected statistically significant temporal trends in species composition. However, these shifts were partly driven by changes in species unrelated to cultural usefulness, and thus cannot directly be interpreted in terms of cultural impact. Moreover, although GAM confirmed significant temporal trends in both NMDS axes, the direction of change was less pronounced than the clear shifts revealed by the usefulness-based ranking. This demonstrates that indicators grounded in cultural use can uncover functionally important ecological changes that standard biodiversity metrics may overlook. Importantly, the long-term vegetation monitoring enabled us to detect these significant changes, underscoring the value of integrating sustained ecological observations with culturally informed indicators. Such an approach underscores the value of combining local knowledge and scientific monitoring to better understand cultural dynamics within SESs.

In this study, we identified CKS by combining interviews with thatch users and the analysis of old thatch samples. One limitation of our study is the small number of interview participants. However, although only two individuals currently use this *kayaba,* they both have long-standing experience in harvesting and using thatch from this area. Their expertise in identifying useful and non-useful species added credibility to the ranking system. Our interpretations were also consistent with earlier interviews conducted with local residents in the region^36^. Additionally, the validity of the interview results was verified by examining the composition of thatch collected from old roofs. These factors support the reliability of the rankings we established in this study. Given the declining number of active thatch users, there is an urgent need to document traditional ecological knowledge (TEK) and associated practices before they are lost. If similar studies are conducted in other *kayaba*, they should be performed promptly while experienced *kayaba* users remain available.

Given these challenges, future research is needed to identify the environmental factors driving the decline in the abundance of useful species and the increase in the abundance of less desirable species. This is essential to clarify what is needed to maintain SES resilience. In addition to water level changes, management practices such as controlled burning influence species composition^35^. Simultaneously, exploring the cultural adaptability of thatch use could prove valuable. For instance, identifying alternative sites for harvesting *shimagaya* and expanding the extent of thatch harvesting, while conserving these sites, would help ensure a stable supply of high-quality thatch. This would support the continued participation of local thatch users and the ongoing transmission of thatching techniques, ultimately enhancing the sustainability of thatch use in this region.

Numerous long-term ecological datasets have been recorded worldwide, reflecting the growing recognition of their importance in understanding ecosystem changes. By integrating such data with humanities-based insights from interviews and sample analyses, a more comprehensive understanding of cultural SES dynamics can be achieved.

Cultural SESs, including those associated with grasslands and wetlands, may be experiencing ecological changes that are not readily captured by conventional ecological assessments. Although this study focused on a specific *kayaba* in Japan, our interdisciplinary approach demonstrates that combining local cultural knowledge with long-term ecological data provides a more comprehensive understanding of resource quality declines that might otherwise go undetected. Applying similar methods to other systems could help identify emerging risks to traditional practices and support the development of more responsive management strategies.

## Conclusion

Although long-term species monitoring has been conducted in many ecosystems, few studies have explicitly linked long-term ecological change to the cultural impacts of such ecological changes on natural-resource-dependent traditions. Here, by creating an interview-based index, we identified looming cultural resource crises that conventional ecological indicators alone would have missed. The observed decline in the quantity of high-quality thatching materials may lead to fewer thatch users, thereby accelerating changes within the SES and potentially triggering an irreversible regime shift.

This study focused on a specific *kayaba*, but the feedback structure in which changes in the quality of natural resources affect their use and, in turn, the continuation of management practices is common to many SESs. While discussions have often emphasized how changes in traditional management or socio-economic and land-use conditions alter ecosystems, our results demonstrate that ecological change itself can reshape management decisions and cultural practices. This perspective is applicable to similar SESs in Japan and worldwide.

For the *kayaba* studied here, future research should focus on identifying and addressing the causes of vegetation change. At the same time, sustained use and management of *kayaba* require robust social support. This includes establishing community-based management systems, fostering trust among stakeholders, and creating mechanisms such as social learning platforms and hands-on programs to engage and increase the number of new participants. These efforts are essential to maintain the cultural SES associated with thatch use and to ensure the continuation of this traditional practice.

Environmental changes, including water regime and climatic factors, may already be causing vegetation shifts in the grasslands used for thatch harvesting in Japan and worldwide. To enhance the resilience of SES and support adaptive responses to cultural resource change, it will be increasingly important to integrate long-term ecosystem monitoring with culturally grounded evaluation. In practice, this integration could involve combining long-term ecological monitoring with the systematic documentation of traditional ecological knowledge, so that changes in biological communities can be analyzed together with cultural practices. The results of such integrative approaches can not only contribute to cultural heritage conservation and policy making but also support the transmission of cultural practices to future generations.

## Methods

### Study site

**Fig. 5:**
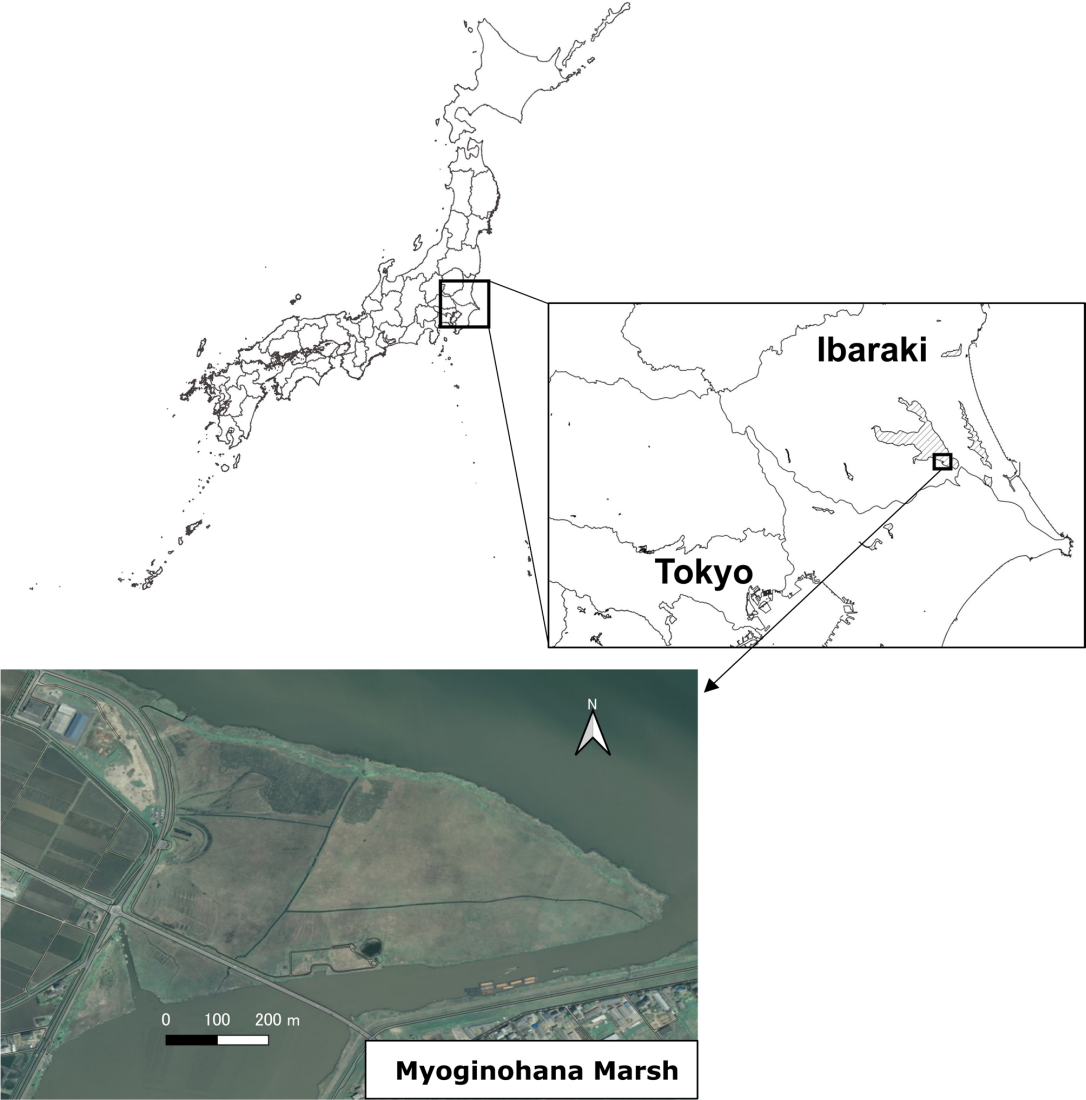
Maps of the study site. Top left, map of Japan; top right, enlargement of the boxed area; bottom, enlarged view of the study area. Aerial photographs from the geospatial information authority of Japan (GSI) were used in the map.

Myoginohana Marsh, our study site *kayaba*, is located on the southern shore of Lake Kasumigaura in Ibaraki Prefecture, near Tokyo (Fig. 5). In this marsh, a type of thatch known as *shimagaya* is harvested. *Shimagaya* does not refer to a specific plant species; rather, it encompasses any thatch made by cutting and bundling various Poaceae plants that grow in this marsh. *Shimagaya* is harvested by using small, walk-behind harvesting and binding machines to create bundles approximately 20 cm in diameter, with no sorting of plant species during the bundling process. This thatch is used in cultural heritage buildings and private residences with thatched roofs. Historical records indicate that it was already being harvested from Myoginohana Marsh by the 1910s^37^, suggesting that thatch harvesting and the associated controlled burning aimed at promoting vegetation regeneration had likely been practiced even earlier. The marsh functioned as a *kayaba* until the 1970s, providing grass for the thatched roofs of local houses. However, as demand for thatched roofs declined, the area used for thatch production gradually decreased. Currently, only one local household harvests *shimagaya* from specific areas in this marsh, between December and January. A thatcher who primarily uses *shimagaya* supports these efforts to help preserve the traditional cultural practice of *shimagaya* harvesting.

In this study, we aim to (1) develop a plant ‘usefulness’ ranking through local knowledge, (2) apply it to 40 years of vegetation data from Myoginohana Marsh to identify long-term trends in thatch quality, and (3) discuss the implications for the sustainability of the thatch-based SES, including management strategies for resilience.

### Semi-structured interviews and observations of thatch samples

First, to clarify changes in the quality of thatch from the users’ perspective, we conducted semi-structured interviews in March 2024 with the only two individuals actively involved in harvesting and using *shimagaya* from this marsh, namely a thatcher and a thatch cutter, who each have decades of experience (about 20 years for the thatcher and more than 60 years for the cutter), and whose knowledge incorporates accounts handed down from earlier generations. In the interviews, we asked about the plant species present within *shimagaya* harvested from the marsh, the usefulness of each species as thatch, and any observed changes in thatch quality (Table 2). Because of the limited number of current practitioners, the number of interviewees was small. To supplement this limitation, we also referred to previous research in which 24 local residents were interviewed in this region between 2006 and 2007^36^, providing additional descriptions of thatch quality and species use. Next, to determine whether the plant species identified as useful in the interviews were actually used as thatch, and to add information on plant species that were not mentioned in the interviews but that may have been utilized, we examined the plant species composition of unused *shimagaya* collected in 2023 and old *shimagaya* (approximately 50 to 100-year-old). Each sample consisted of one bundle (approximately 20 cm in diameter). The old thatch was taken from the roof of a cultural property temple in the study region during roof repairs. Harvested and bundled thatch is transported directly to the roof without prior selection, and unnecessary plants are removed during the thatching process. Therefore, unused thatch contains both plants that are used for roofing and those that are discarded. In contrast, old thatch retrieved from roofs has already undergone this selection process and therefore consists only of plants that were deemed suitable for use as thatch.

On the basis of the interviews and sample observations, plant species were categorized into four groups from the perspective of thatch usefulness: Rank A, Species useful as thatch; Rank B, Species that are used without being removed when bundled together with Rank A species; Rank C, Species that can be mixed in small amounts in bundled thatch but that become unusable in large proportions; and Rank D, Species that must be removed because they clearly reduce the thatch quality (Table 1).

### Analysis of vegetation survey data for Myoginohana marsh

Vegetation surveys have been conducted in Myoginohana Marsh by the Japan Water Agency for more than 40 years^38^. The survey involves vegetation mapping and flora surveys based on phytosociological community composition. Flora surveys were conducted in 1981 and annually from 1996 to 2021, while vegetation maps were produced annually from 1996 to 2021. We used these data to analyze changes in the availability of thatch materials in the marsh.

First, to analyze the changes in the area dominated by useful plants, vegetation maps of the marsh from 1996 to 2021 were analyzed. As each plant community is named on the basis of its dominant species, we assigned these dominant species a rank of usefulness for thatch collection. By using these ranks, we then calculated changes in the area dominated by useful plants across the entire marsh.

Secondly, to examine changes in species composition and quality, flora surveys conducted in the fall (September to November) from 1981 and annually from 1996 to 2021 were analyzed. The years 1982 to 1995, 2000, and 2002 were excluded because of data unavailability. The surveys recorded the species cover within 1m^2^ quadrats, assessed by using the Braun-Blanquet method^39^. The scale of this vegetation survey was roughly equivalent to the composition and proportion of plants in a bundle of thatch, so we assumed that the results would reflect the species composition of thatch bundles. Based on interview results indicating that the thatch cutter specifically targeted areas where *Ischaemum aristatum* var. *glaucum* grew, we restricted the analysis to quadrats containing this species to ensure that we were examining the parts of the marsh relevant to *shimagaya* harvesting. The survey months, quadrat numbers, and survey locations varied each year. The cover classes recorded by using the Braun-Blanquet scale were converted into percentages and their median values were used in the analysis (87.5% for 5; 62.5% for 4; 37.5% for 3; 17.5% for 2; 5.5% for 1; and 0.5% for +). Both conventional vegetation analysis methods and cultural-perspective-based analysis were conducted. For the conventional methods, the species richness and the Shannon–Weaver diversity index (*H’*) per quadrat were obtained. To assess temporal changes in species composition, we conducted a non-metric multidimensional scaling (NMDS) ordination based on Bray–Curtis dissimilarity of species cover values. Cover data were square root transformed prior to analysis to reduce the disproportionate influence of dominant species. The number of dimensions (k) was set to 2, and the robustness of the solution was confirmed by comparing runs with different numbers of random starts (trymax = 50 vs. 100) as well as between two- and three-dimensional solutions. Procrustes tests showed that the two-dimensional (k = 2) solutions were identical across trymax settings, and that the discrepancy between two- and three-dimensional solutions was minimal (RMSE = 0.0023). We therefore adopted the two-dimensional solution with trymax = 50 for analyses.

Temporal trends were analyzed by using a generalized additive model (GAM) with cubic spline smoothing for year, implemented in the mgcv package in R. This approach is well suited for detecting long-term patterns in datasets with irregular sampling, such as variation in survey months, locations, and sample sizes across years.

Thirdly, as the height of *I. aristatum* var. *glaucum* was identified as an important factor in thatch quality and was reported in interviews to have become shorter in recent years, we compared the species’ height between 1981 and 2023. In the 1981 survey conducted by the Japan Water Agency, 1-m^2^ quadrats were set up within areas where *shimagaya* was harvested every year, and the representative height of plant species within the quadrats was measured monthly from June to November. For comparison with these data, we conducted a separate survey in 2023 by using the same method. Quadrats were established in areas where *I. aristatum* var. *glaucum* was growing, and the representative height was recorded monthly from June to November. On the basis of the results of *F*-tests, paired-sample *t-*tests (two-sided, at a 5% significance level) were performed for October, whereas unpaired-sample *t*-tests (two-sided, at a 5% significance level) were conducted for the other months. Statistical analyses were performed by using R version 4.2.2.

### Ethical considerations and informed consent

This study did not involve sensitive personal data or vulnerable populations, and no identifiable personal information was collected. The interview protocol was designed and conducted in accordance with the Japanese Government’s *Ethical Guidelines for Life Science and Medical Research Involving Human Subjects* and the University of Tokyo’s *Research Ethics Regulation*. Participants were fully informed about the purpose of the research and their rights, and gave informed verbal consent to participate and to allow their anonymized responses to be used in academic publications.

The research protocol, including the interview procedure and informed consent process, was submitted to the Research Ethics Committee of the University of Tokyo. The Committee reviewed the study and approved the exemption from ethical review under the same institutional and governmental guidelines. The study was conducted in accordance with the principles of the Declaration of Helsinki.

## Supplementary Information

Supplementary Information.

## Data Availability

The vegetation survey dataset used in this study was provided by the Japan Water Agency and is not publicly available, but can be requested directly from the agency. The code used for the quantitative analyses is available from the corresponding author upon reasonable request.
