# Supplementary material for: Vegetation change threatens the sustainability of cultural keystone species and traditional ecological knowledge in a social–ecological system
Source: Sci Rep. 2025 Nov 17;15:40308. doi: 10.1038/s41598-025-24015-6 (PMC12623805; doi:10.1038/s41598-025-24015-6)
Supplement: Supplementary file 1 — Supplementary Information. [file 41598_2025_24015_MOESM1_ESM.pdf]

Supplementary Information

Title:

Vegetation Change Threatens the Sustainability of Cultural Keystone Species and Traditional Ecological Knowledge in a Social–Ecological System

Authors:

Shiori Takahashi*[1], Jun Nishihiro[2]

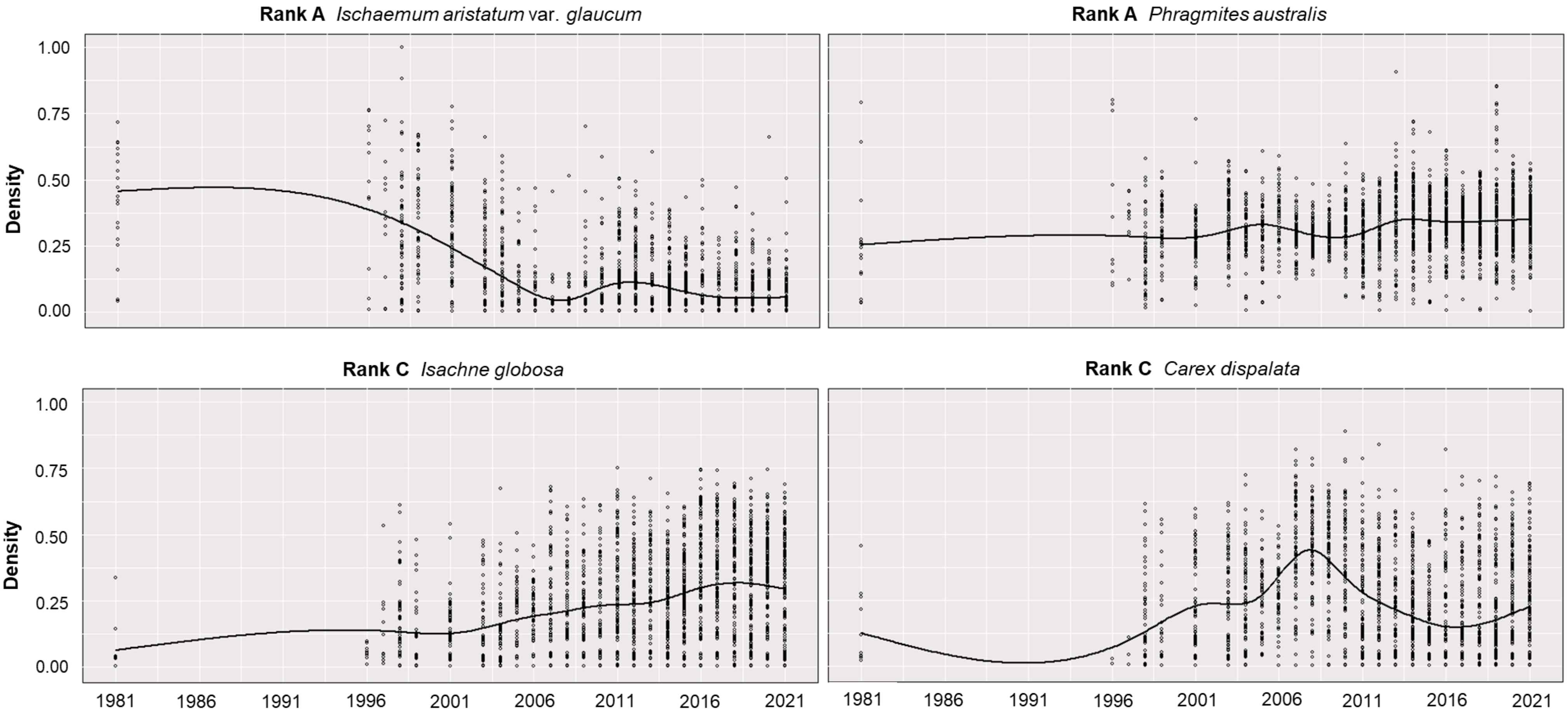

Supplementary figure 1. Temporal changes in the coverage percentages of representative species with high coverage within Rank A and Rank C, as analyzed by using a generalized additive model.

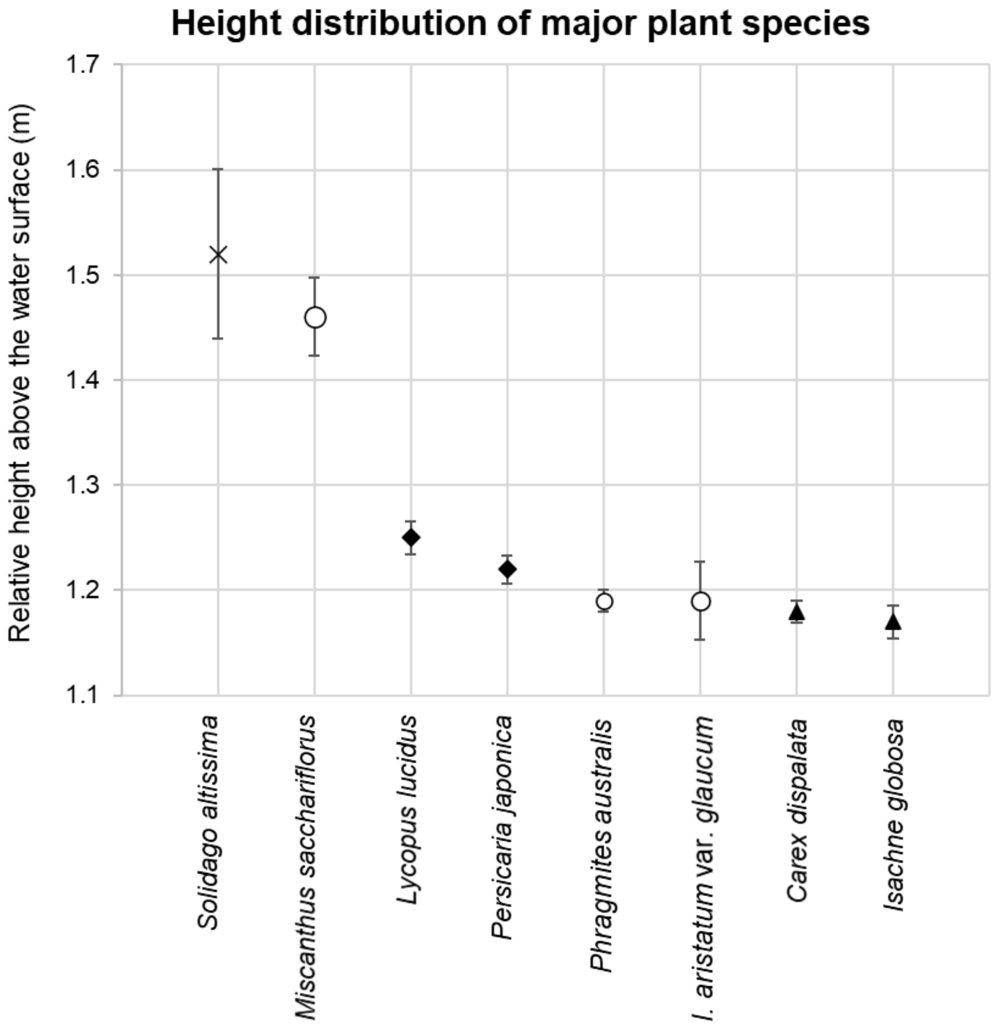

Supplementary figure 2. Heights above the water surface where major thatch species grow in Lake Kasumigaura. Markers in the graph indicate the rank of each species: ○, Rank A; ◆, Rank B; ▲, Rank C; ×, Rank D.

The number of samples for each species was as follows: *Solidago altissima*: n = 50; *Miscanthus sacchariflorus*: n = 41; *Lycopus lucidus Turcz*: n = 119; *Persicariajaponica*:

n = 151; *Phragmites australis*: n = 190; *Ischaemum aristatum* var. *glaucum*: n = 14; *Carex dispalata*: n = 270; *Isachne globosa*: n = 13.
